# Metal and Metal Oxide Nanoparticle as a Novel Antibiotic Carrier for the Direct Delivery of Antibiotics

**DOI:** 10.3390/ijms22179596

**Published:** 2021-09-04

**Authors:** Harshada Kotrange, Agnieszka Najda, Aarti Bains, Robert Gruszecki, Prince Chawla, Mansuri M. Tosif

**Affiliations:** 1 Department of Food Technology and Nutrition, Lovely Professional University, Jalandhar 144411, Punjab, India; harshada.k21@gmail.com (H.K.); tosifmansuri444@gmail.com (M.M.T.); 2 Department of Vegetable Crops and Medicinal Plants, University of Life Sciences in Lublin, Doświadczalna Street, 20-280 Lublin, Poland; robert.gruszecki@up.lublin.pl; 3 Department of Biotechnology, CT Institute of Pharmaceutical Sciences, South Campus, Jalandhar 144020, Punjab, India; aarti05888@gmail.com

**Keywords:** nanotechnology, metal nanoparticle, antimicrobial activity, resistance, bacteria

## Abstract

In addition to the benefits, increasing the constant need for antibiotics has resulted in the development of antibiotic bacterial resistance over time. Antibiotic tolerance mainly evolves in these bacteria through efflux pumps and biofilms. Leading to its modern and profitable uses, emerging nanotechnology is a significant field of research that is considered as the most important scientific breakthrough in recent years. Metal nanoparticles as nanocarriers are currently attracting a lot of interest from scientists, because of their wide range of applications and higher compatibility with bioactive components. As a consequence of their ability to inhibit the growth of bacteria, nanoparticles have been shown to have significant antibacterial, antifungal, antiviral, and antiparasitic efficacy in the battle against antibiotic resistance in microorganisms. As a result, this study covers bacterial tolerance to antibiotics, the antibacterial properties of various metal nanoparticles, their mechanisms, and the use of various metal and metal oxide nanoparticles as novel antibiotic carriers for direct antibiotic delivery.

## 1. Introduction

Microbial infections and contamination are persisting as the foremost cause of morbidity and mortality in hospitals across the globe [1]. However, antimicrobial components and therapy act as a milestone in medicine, by saving millions of lives [2]. Despite the availability of several broad-spectrum antimicrobial components, antimicrobial resistance is a worldwide health emergency, and it is an enormous challenge for the effective therapy and remedy of many common infections [3,4]. Resistance to antimicrobial components can be defined as the ability of a disease-causing microorganism to resist the therapeutic effects of drugs [5]. As a result, standard treatments become useless, and contaminations persist and may spread to others. Antimicrobial resistance, developed among clinical and environmental microorganisms, has become a widely spread phenomenon, which has already been recognized by national and international regulatory authorities. Antibiotic resistance is a narrower term, since it refers to the resistance to drugs that treat infections that are caused by bacteria [6]. However, each year, more than two million people are suffering from infections of antibiotic resistance; it is believed that the global molarity rate will exceed 10 million annually by the year 2050 [7]. DNA replication machinery, translational machinery, and cell wall synthesis are the three main targets of antibiotics that are currently in application. Consequently, bacterial resistance to each of these mechanisms of action can exist. Besides, several commonly used antibiotics, such as ceftazidime/avibactam (against KPC-producing *Klebsiella pneumoniae*), daptomycin (against *Staphylococcus aureus*), caspofungin (against *Candida*), fluconazole (against Candida), and penicillin (against *Staphylococcus aureus*), have developed resistance in the human body [8,9]. Therefore, nanoparticles (NPs) can be promising drug delivery vehicles that have the potential to solve present medicinal technical challenges, due to their better bioavailability, lower toxicity, controlled release, and specific targeting [10]. NPs are colloidal nanoparticles that are utilized for a variety of purposes, including the delivery of antibiotics. They are particles with a diameter of less than 1000 nanometers that are used in nanomedicine [11]. Also, they have high antibacterial, antifungal, antiviral, and antiparasitic activity. Antibiotic delivery through nanoparticles, to the site of infection, is a potential therapeutic approach, especially for controlled drug release, which reduces the amount required to produce a clinical effect [12]. Moreover, hydrogels, metal colloids, dendrimers, polymers, and lipids are significantly used for synthesizing NPs [13]. The antimicrobial mode of action of NPs can be classified into the following three categories: non-oxidative, metal ion release, and oxidative stress induction processes. Among all the delivery vehicles, metallic nanoparticles, such as silver (Ag), gold (Au), copper (Cu), iron (Fe), and zinc (Zn), have attracted the attention of scientists all over the world, due to their growth potential in drug delivery [14]. Metal nanoparticles (MNPs) have been identified to create unspecific microbial resistance pathways that not only inhibit bacterial tolerance growth, but also expand the range of antimicrobial activity. Furthermore, the lower hydrophilicity of MNPs induces aggregation, which inhibits their applicability, especially in biological treatments. Several studies revealed that silver nanoparticles (AgNPs) induce the electric charge of the bacterial membrane’s surface to be neutralized and its penetrability to change, resulting in bacterial killing. Furthermore, the production of reactive oxygen species (ROS) inhibits the antioxidant defense mechanism and damages the cell membrane functionally [15,16]. The key pathways behind the antibacterial actions of MNPs, according to existing studies, are as follows: (i) disruption of the bacterial cell membrane; (2) production of reactive oxygen species (ROS); (3) penetration of bacterial cell membrane; and (4) development of intracellular antibacterial effects, including interactions with DNA and proteins [17]. Metal oxides are formed as metal ions form bonds with oxides, resulting in a closely packed structure. MO plays an important part in material science and some other areas [18]. Therefore, the current review emphasized bacterial resistance to antibiotics, the mechanism of bacterial resistance, and the antibacterial properties of various metal nanoparticles. Also, the use of various metal nanoparticles as novel antibiotic carriers for direct antibiotic delivery is discussed with a mechanism and schematic diagram.

## 2. Bacterial Resistance against Antibiotics

Antibiotics are, without dispute, the most effective type of chemotherapy, and since they were first commercially available, they have extended life expectancy by up to two decades. However, over the last few decades, dangerous antibiotic-resistant bacteria have become increasingly common. Antibiotic resistance has been a known reality almost since the beginning of the antibiotic period, but it has only been in the last two decades that harmful, resistant strains have emerged with depressing regularity [19]. Antibiotic resistance (ABR) has emerged as a significant challenge to human health in the 21st century. ABR is expected to cause 10 million deaths globally by 2050, and to push 24 million people into deep poverty by 2030. Antibiotic-resistant infections were shown to be the most common in children under the age of one and older people over the age of 65. Antibiotics have been used indiscriminately for decades, resulting in the growth of multidrug resistance. Antibiotic resistance concerns global health regulatory bodies, and researchers are working around the globe to solve the problem [20]. In addition to the effects, increasing the constant need for antibiotics has resulted in the growth of antibiotic bacterial resistance over time. Antibiotic tolerance mainly evolves in these bacteria by efflux pumps and biofilms [21]. Furthermore, considering the steady growth of the production of new antimicrobial agents, and the fact that most health-related policies aim to implement an efficient treatment prevention policy, long-term investment in combating antimicrobial resistance is essential. Nanoparticles have recently been shown to be very effective against antibiotic-resistant pathogenic bacterial strains that are clinically important [22]. Innate and acquired antibiotic resistance are the two forms of resistance. By contrast, innate immunity, which offers germline-encoded immediate protection for the host from infections, has retained its antimicrobial effectiveness for millions of years, with no frequent emergence of resistant strains [23]. Innate resistance, also known as natural resistance, is a type of resistance that is normally gained by microorganisms. Antibiotics cannot reach the targets, since the microorganism’s cell wall lacks target areas for antibiotic binding [24]. Moreover, acquired antibiotic resistance is when a pathogen that has historically been immune to infections gains immunity, and due to a genetic variant or lateral genetic transformation, susceptibility develops [25]. Some pathways combine to give a high immunity to a particular drug in some situations [26].

### 2.1. Mechanisms of Bacterial Resistance againts Antibiotics

**Figure 1:**
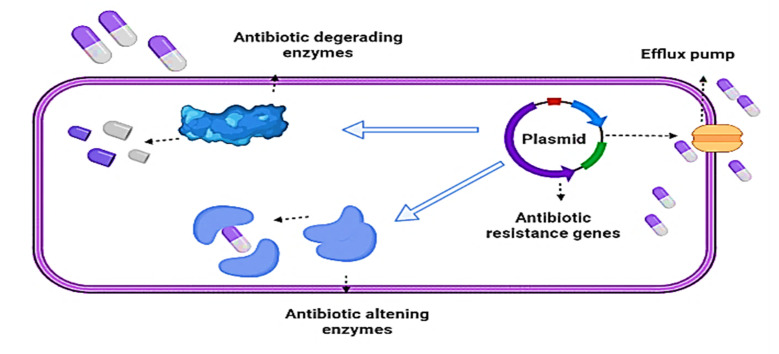
Mechanism of bacterial resistance to antibiotics.

Antibiotics have been used extensively in recent decades to treat a variety of diseases caused by bacterial infections, but this has resulted in an increase in antibiotic resistance among bacteria. Obtaining antibiotic resistance genes (ARGs) by gene mutation or horizontal gene transfer is one of the most significant factors for bacterial resistance. Antibiotic selection pressure on bacteria encourages the growth and spread of ARGs [12]. Many resistant bacteria, bearing many ARGs, have been discovered in environmental factors, such as water, air, soil, sediments, and also in humans and livestock, making disease detection and management more complex and creating a significant health risk. As a result, finding new possible antibiotic alternatives to limit ARG contamination is critical [27]. Drugs are becoming resistant to bacteria by following several pathways, such as aggregation, target binding, and downstream toxicity. Genomic modifications, ranging in size from point mutations to preexisting genetic elements assembly to horizontal gene import from the atmosphere, encode these pathways; bacteria have also evolved a complex system of tolerance to antimicrobial agents to survive. Antimicrobial resistance is acquired by bacteria via a variety of biochemical mechanisms, including gene mutation encoding the target antimicrobial site, efflux overexpression resulting in antibiotic exclusion from the cell, and target site protection through proteins (Figure 1) [28]. 

The main mechanisms for antibiotic resistance (efflux pump, antibiotic inactivation, target site change, modification/elimination of antibiotic entry points) are enzymatic (β-lactamases) and non-enzymatic susceptibility pathways. After biofilm formation, these pathways may still function in the single cell. Exopolysaccharides or other extracellular polymeric molecules (extracellular DNA, amyloid fibers, etc.) are found in biofilms, as well as molecules derived from the host, such as mucus and DNA. Antibiotic entry into resident bacterial cells is limited by biofilms [29].

### 2.2. Enzymatic Mechanisms

#### β-Lactamases Production

The enzyme -lactamase is mostly responsible for β-lactam drug resistance. These enzymes are hydrolyzing enzymes that break the amide bonds within the β-lactam ring, causing antimicrobial agents to affect [30]. In 1940, scientists discovered the use of antibiotic penicillin–-lactamases enzymes. Within the amber class, these enzymes are divided into four groups (A, B, C, and D) based on the amino acid composition. Class A carbapenemases are classified into five classes, three of which are chromosomally encoded (imipenem-hydrolyzing enzyme (IMI), S. marcescens enzyme (SME), and non-metalloenzyme carbapenemase (NMC)), and two of which are plasmid-encoded (KPC and GES) [31]. More than 50 years ago, non-pathogenic bacterial chromosomal genes were discovered to contain class B enzymes. These enzymes were discovered in pathogenic strains of Pseudomonas aeruginosa, Enterobacter spp., and Acinetobacter spp. later in the 1990s. Both cephalosporins and penicillins are immune to class C lactamases. These enzymes are not hydrolyzed by aztreonam and are immune to clavulanic acid [32]. CHLDs, also known as oxacilinases (OXA), because of their ability to oxidize oxacillin, contain serine with an inactive catalytic domain, and are present in the two functional groups Bush and Jacoby. Many important Gram-negative pathogens are responsible for producing widespread class B β-lactamases, such as *K. pneumoniae* carbapenemase (KPC) and New Delhi metallo-β-lactamase (NDM) that are known to be an ultimate resort for the treatment of infections in chronically ill patients, for example, imipenem, meropenem, and ertapenem. Current inhibitors, such as clavulanic acid 1, sulbactam 2, or tazobactam 3, do not adequately inhibit KPC. This is due to the special arrangement, which is distinguished by a massive active site, which can handle bulkier β-lactams as opposed to other class A β-lactamases [33].

### 2.3. Non-Enzymatic Mechanisms

#### 2.3.1. Efflux Pump

In Gram-positive and Gram-negative pathogens, the efflux pumps constitute one of the most effective antibiotic resistance mechanisms through which cell microorganisms are aggressively dehydrated. Furthermore, the resulting sublethal concentration of medicines may contribute to the acquisition of a different mechanisms of resistance linked to mutations. Multidrug treatment antibiotic resistance has been found in efflux pumps. Both species, including prokaryotes, eukaryotes, and human cells, have transporters. AcrAB222 TolC is an efflux pump that belongs to the RND family, and it is one of the most studied efflux pumps in *E. coli* [34]. From drug entry through aggregation and target binding to downstream toxicity, resistance mechanisms work to stop the drug. The prevention of unknown entrance of the drug into the cell is the outermost defensive line. A difference in the chemistry or bacterial cell thickness may prevent antibiotics from spreading into the cell (permeability). Cell membranes can often carry drugs or general pumps (efflux pump). Bacterial resistance to macrolide is another example of efflux-mediated resistance, which was first found in Gram-positive bacteria, such as Staphylococcus aureus [35].

#### 2.3.2. Inhibitors of Bacterial Biofilm Formation

Biofilms are bacterial communities with a high degree of multifactorial tolerance. Antibiotics that are effective against planktonic cells are ineffective against biofilms. Many efforts have been made in the last decade to find new antibacterial agents that are suitable for treating biofilm-associated infections [36]. Antimicrobial agents operate by the following three major action mechanisms: the first step of the bacterial pathogens is to suppress adhesive bacteria to surfaces; (ii) the biofilm architecture is distorted in the maturation process; and (iii) a quorum-sensing system interferes. β-lactamases, chloramphenicol amino glycoside-modifying enzymes, and acetyltransferases (AACs) are three main enzymes that inactivate antibiotics [37].

#### 2.3.3. Modification of the Target Site

Some examples of modification are a change in the target (residue substitution), target binding by a protective factor, or change in target abundance. Notably, although the over-expression of certain medicines raises the resistance of such products, for some medications the resistance may be decreased. In the end, the final protection line can be poisonous, to prevent the impact of target binding, by eliminating the need for the chemical reaction in which the target is affected, or by modifying the chemical structure and function of the cell (metabolic shunt) [18]. It is intended to change or, in some situations, degrade drug chemical compounds. This form of modification is common in bacterial isolates that result in drug resistance to a wide variety of drugs, regardless of the molecular mechanism [22].

#### 2.3.4. Modification/Elimination of Antibiotic Entry Points

Antibiotics are killed by yet another bacterial process. It is intended to change or, in some situations, degrade drug chemical compounds. This form of modification is common in bacterial isolates that result in drug resistance to a wide variety of drugs, regardless of the molecular mechanism [38]. The following line of protection stops the aggregation of drugs by chemical targeting (modification of medicine—accumulation): assigned enzymes to alter or hydrolyze drug molecules (degradation). These reactions may occur inside the cell or, whether the enzymes are secreted, preventably outside the cell. Even if medicines are accumulated in the unmodified bacterial cytoplasm, a target shift (target alteration—binding) can impede their binding and inhibit the targets. Bacterial strains contain enzymes that catalyze and attach particular chemical fractions to antimicrobial substances, allowing them to be made inactive. These enzymes can destroy molecules or damage antibiotics by interacting with the target sites on the microorganism membrane on their own [39].

## 3. Synthesis of Metal Nanoparticles (MNPs) Complex for Delivery of Antibiotics

**Figure 2:**
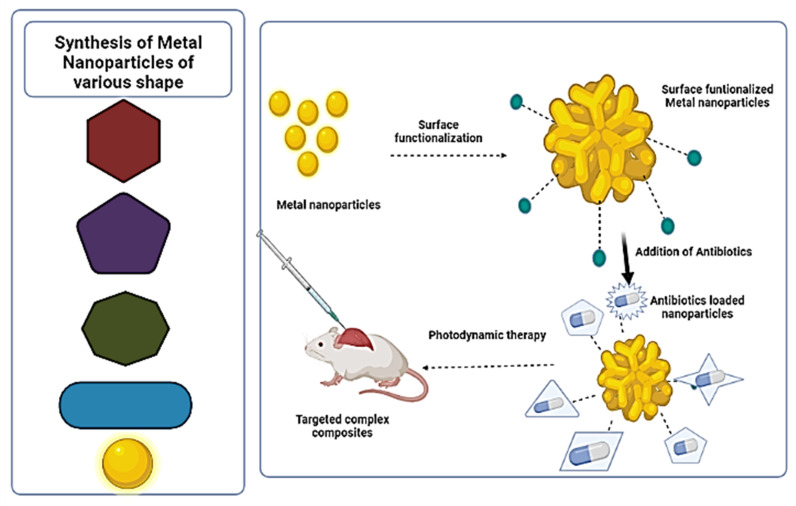
Different shape and size metal and metal oxide nanoparticles can be synthesized by different methods. This scheme represents surface functionalized by the different ligands, enzymes, polymeric components, which improves the binding sites of nanoparticles. Functionalized nanoparticles and antibiotic complex can be directly introduced to target test animal.

Over the past years, nanotechnology, such as cellular or molecular biology, semiconductor technology, and information technology, is expected to have a significant impact on society and our economy [40]. Moreover, national security, information technology, energy, biotechnology, medicine and healthcare, nanoelectronics, and materials and manufacturing are all areas where nanotechnology research promises breakthroughs. Furthermore, there are several chemical methods of the synthesis of MNPs that have been developed, such as the biochemical method, the microemulsion method, irradiation reduction, microwave-assisted synthesis, photo-catalytic reduction or photo-induced, ultrasonic-assisted reduction, electrochemical reduction, the template method, nonaqueous chemical reduction, aqueous solution chemical reduction, and chemical reduction [41,42]. However, these chemical processes have been associated with numerous disadvantages, including excessive energy consumption, the production of hazardous by-products, and the use of toxic solvents, all of which offer serious health and environmental hazards. Metal nanoparticles are being used as targeted delivery of drugs, by surface functionalization. However, metal nanoparticles are surface functionalized with the combination of antibiotic drugs, as shown in Figure 2. Herein, metal nanoparticles can be synthesized in various shapes. In this context, Seyed-Talebi et al. [43] synthesized the TiO_2_ hollow spheres by a green, easy and cheap process for the direct delivery of gentamicin. Carbonaceous spheres were used as the removable template. The result of the study proved that prepared TiO_2_ hollow spheres have excellent antibiotic carrier properties for the direct delivery of gentamicin. Surface and interaction between gentamicin, due to their spherical morphology, more abundant porous structure, and larger specific surface area.

Furthermore, there is a rising demand to develop an environmentally friendly nanoparticle synthesis method that does not need the use of harmful chemicals. Therefore, the biological synthesis of MNPs by microbes has recently been suggested as a significant source of nanomaterials mining [44]. The microbial recovery (bacteria, fungi, yeast, and virus) of precious metals, through the creation of nanoparticles, is a more environmentally friendly way than the traditional method. Similarly, AgNPs biosynthesis using plants, fungi, and bacteria has been extensively reported. Many active groups in antibiotic compounds, such as amide and hydroxyl groups, react easily with AgNPs via chelation, resulting in efficient resistance [45,46]. In this context, Gandhi and Khan [47] synthesized the silver nanoparticles (AgNPs) from the Gram-negative (Escherichia coli) bacterial cells. Further, these AgNPs were coated on antibiotics and investigated for delivery. Moreover, the synthesized complex of AgNPs with antibiotics against the Gram-positive and Gram-negative bacteria offer a significant contribution, with great potential as a delivery vehicle to nanomedicine. Also, the result of the study revealed that the antibacterial activities of ampicillin, kanamycin, gentamycin, streptomycin, and bacitracin were increased in the presence of silver nanoparticles against the test strains. Besides, the shape and size of the NPs for targeted drug delivery would inevitably have managed to improve the drug carrier efficiency, due to its adverse effects on in vivo particle marginalization, by interactions with a different cell. According to several studies, novel antibiotic compositions in nanoparticle structures may increase therapeutic potency, implying that medication combinations containing drug-loaded nanocomposites may have a safer treatment outcome for infections requiring large multidrug concentrations. Fe_3_O_4_ nanoparticles that are smaller than 5 nm can penetrate the nucleus, while those larger than 9.5 nm can only be found in the cytoplasm. Owing to the excellent tendency of the nucleus to bind, metal oxides between 2.1 nm and 4.3 nm have a greater potency than 9.5 nm nanoparticles [48,49,50]. Consequently, the complex of MNPs, such as ZnO and Ag, were synthesized along with antibiotic combination against the microorganisms, such as *C. albicans, E. coli, Salmonella enterica subsp. Bukuru, and Staph. aureus,* by Khurana et al. [51]. Herein, *Ulva fasciata* alga were used as the biological synthesizer for MNPs. The result of study proved that the synergistic effect of the exanimated antibiotics, such as chloramphenicol, fosfomycin, cefuroxime, cefotaxime, and azithromycin, against the pathogenic bacteria (*E. coli*), was increased in the presence of silver nanoparticles, as compared to the antibiotic only. 

## 4. Characterization of Metal Nanoparticles (MNPs) Complex

**Figure 3:**
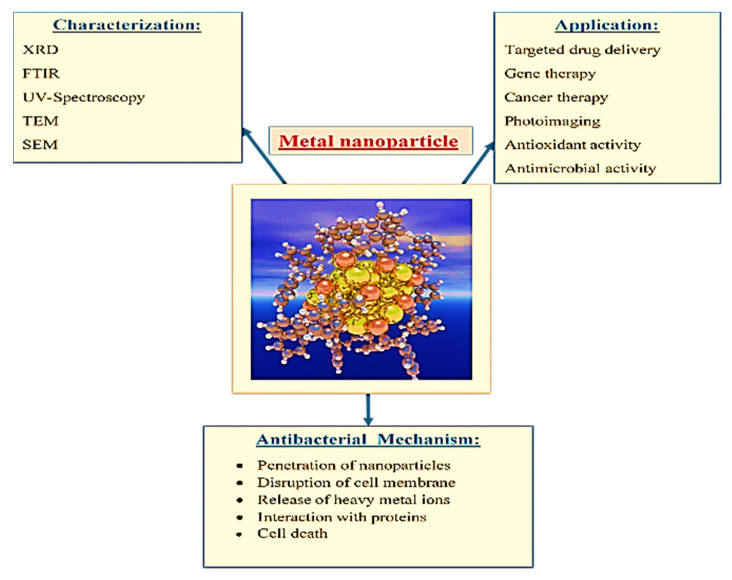
Characterization of metal nanoparticles using different instrumental-based techniques.

The characterizations of chemically or biologically synthesized nanoparticles can be conducted by several techniques, such as FTIR, SEM, TEM, XRD, etc. (Figure 3). Similarly, Roshmi et al. [52] investigated the FTIR of metal nanoparticles that were functionalized with antibiotics. Except for rifampicin, all the antibiotics displayed a characteristic absorption band at 1600–1635 cm^−^^1^, which corresponded to the N–H bending frequency. The N–H bending peak was moved to a higher wavelength in the case of antibiotic-coated gold nanoparticles. Due to the symmetrical and asymmetrical stretching of the methylene ring, rifampicin had an absorption band at 2922 and 2939 cm^−1^, which was moved to 2949 in the rifampicin–AuNPs conjugate. Furthermore, in the IR spectrum of rifampicin-coated gold nanoparticles, an N–H bending peak at 1645 cm^−1^ was observed.

Likewise, Assadi et al. [53] characterized hyperbranched polyglycerol-coated copper oxide nanoparticles by the FTIR technique. The result of FTIR confirmed the formation of CuO, and corresponded to the Cu-O stretching mode with peaks at 1374, 595, 497, 438 cm^−1^. Moreover, the wide peak was observed at 3433 cm^−1^, which determined the stretching vibrations of O–H, and an absorption peak (at 1623 cm^−1^) confirmed the O–H bending (adsorbed water combined with Cu molecules). The strong peak of the FTIR spectra of copper oxide nanoparticles with hyperbranched polyglycerol was at 3400, 1100, and 2900, attributed to the O–H, C–H, and C–O–C stretching modes, respectively. FTIR concluded the conformation of strong modification and polymerization onto the copper oxide nanoparticles, by hyperbranched polyglycerol. Consequently, UV/Vis spectroscopy was used to monitor improvements in surface plasmon resonance (SPR), as the solution was incubated at room temperature with gentle magnetic stirring [54]. IR amoxicillin-bound amoxicillin spectrums were identified and zeta potential calculation assessed the surface burden of gold nanoparticles before and after the loading of amoxicillin. To confirm the drug nanoparticle interaction, XRD measurements were taken [55]. The nanoparticles of zinc oxide have been synthesized in an alkaline state by a chemical process. A sharp peak at 390 nm, with UV–Vis spectroscopy, confirmed the initial formation of single-scattered nanoparticles with zinc oxide. Also, the formation of multi-formed zinc oxide nanoparticles, with an average size of 20 to 24 nm, have been developed by microscopic techniques, such as SEM and TEM. The UV–Vis result revealed an intense peak at 540 nm, which corresponded to the gold nanoparticles’ surface plasmon band, according to Roshmi et al. [52]. Besides, the narrow peak also revealed the monodispersed nature of the produced gold nanoparticles in solutions. After a year of storage at 4 °C, the stability of the gold nanoparticles was investigated using UV–Vis spectroscopy. Even after this time, the color of the gold nanoparticles solution and absorption peaks remained unchanged, suggesting that biosynthesized gold nanoparticles are stable. This could be because gold nanoparticles are coated with biomolecules during synthesis, neutralizing the electrostatic force around them and preventing aggregation. Similarly, Ashfaq et al. [56] investigated the surface morphology of bimetal nanoparticles (Cu and Zn) dispersed with carbon nanofibers, by scanning electron microscopy (SEM). The high-magnification SEM pictures revealed the shiny metal nanoparticles at the tip of the carbon nanofibers. However, in the midst of the fibers, there were only a few metal nanoparticles. Carbon nanofibers grew irregularly or in a relatively poor manner in the case of antibiotics and metal nanoparticle samples [57]. Moreover, Singh et al. [27] synthesized the silver nanoparticles from *Acinetobacter calcoaceticus* combined with antibiotics. The XRD result showed the four peak valued of 77.2°, 64.4°, 44.3°, and 38.1°, at 20, corresponding to 311, 220, 200, 111, respectively. Similarly, the SEM results of the study showed the nanoparticles formations in aggregated and dispersed forms; these were all confirmed to be of silver by EDS. A copper grid was used for the EDS analysis, which was main reason for the additional peaks for copper.

## 5. Antimicrobial Activity of Metal Nanoparticles and Antibiotics Complex

**Figure 4:**
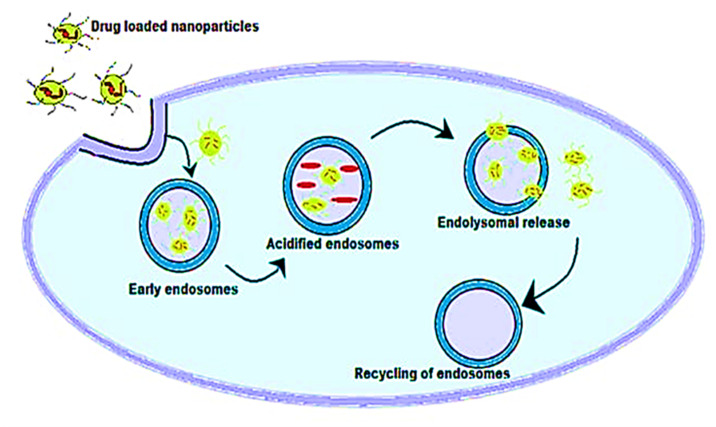
A schema of nanoparticle internalization in the cells.

Metal nanoparticles (MNPs) are excessively used in various industrial applications, such as technology, scientific researches, and development, due to their advantageous properties. Metal oxides work as antibiotics by releasing metal ions into the bacterial cell, where they interact with nucleic acid functional groups and proteins. This contact changes the structure of the cell, inhibits enzyme activity, and disrupts the bacterial cell’s physiology [12]. In this context, in a study conducted by Bello-Vieda et al. [58], silver nanoparticles were used to evaluate the antimicrobial activity against Gram-negative and Gram-positive pathogenic bacteria. When silver nanoparticles (AgNPs) were complexed to either sulfated or carboxylated NPs, the MIC values of some bacterial strains were greatly reduced (P 0.05 by ANOVA) in comparison with the controls, *Pseudomonas aeruginosa* (ATCC 27853), *Proteus vulgaris* (ATCC 33420), *Salmonella typhimurium* (ATCC 13311), and *Klebsiella pneumonia* (ATCC 27736), respectively. For both Gram-positive and Gram-negative bacteria strains, the association of AgNPs with metal complexes demonstrated bactericidal effects at lower concentrations than in the absence of AgNPs. When antibacterial actions were mixed with AgNPs at a steady concentration of 2 g mL^−1^, the azole concentration was reduced up to 10 times. The azoles and AgNPs’ synergistic influence did not affect HFF (human foreskin fibroblast) cellular viability. The complexes in combination with AgNPs demonstrated promising antibacterial activity against a variety of bacterial strains [59]. Moreover, nanoparticles were prepared from chitosan with a molecular amoxicillin. The result showed that amoxicillin inhibits the development of *S. pneumoniae*. As compared to amoxicillin alone, the antibacterial efficacy of the chitosan nanoparticle–amoxicillin complex improved significantly. This means that the chitosan nanoparticle–amoxicillin complex has a three-fold lower MIC performance against *S. pneumonia* than amoxicillin alone [60]. The role of silver in the complex may be responsible for the antibacterial efficacy of clotrimazole–Ag as opposed to clotrimazole. Several studies proved that there are the following two modes of action of silver: First, the high binding avidity of the adverse side groups, which is scattered through the microbial cells (carboxyl, phosphate, sulfhydryl, and others), transforms the molecular structure and makes this unusable to the cell. Second, it can target various cell sites that interact with protein folding and functioning, membrane processing, cell wall synthesis, nucleic acid synthesis, translation, transmission, and the transport of electrons. This inhibits the development of bacteria, which kills the bacteria and indicates that silver tolerance is extremely unlikely to evolve [61]. The silver–furosemide complex (Ag-FSE) has been encapsulated with ~93% EE in solid lipid nanoparticles (SLNs). Studies in in vitro releases have verified the continuous release of Ag–FSE in SLNs for 96 h. The results confirmed that the behavior of *P.aeruginosa* and *S. aureus* was a two-fold and four-fold improvement. Ag–FSE nanoparticles from solid lipids may be regarded as an advantageous topical antibacterial against bacterial inflammation [62]. The involvement of AgCl NPs and silver ions in Ag–AgCl@C11-mPEG NPs demonstrates antimicrobial activity against Gram-positive bacteria. At the same time, only 5/1 of the red NPs shows Gram-negative activity that is intrinsic to the behavior of Ag NP. Also, in contrast with other Ag–AgCl@C11-mPEG NPs, the highest overall activity is observed with all the studied pathogenic bacteria [63]. A schema of nanoparticles internalization in the cells is explained in Figure 4.

Zinc oxide (ZnO) is a desirable metal oxide nanoparticle, due to its broad range of applications in areas such as optical, gas sensing, magnetic, piezoelectric, water treatment, and disinfectants [64]. Zinc oxide nanoparticles (ZnO-NPs) have attracted a lot of research attention because of their wide range of applications in smart ultraviolet sensors, selective gene therapy, antibiotic work, environmental remediation of biosensors, and also as an aspect improving drought resistance and seed availability of nutrients [65]. ZnO nanoparticles have piqued researchers’ interest among metal oxide nanoparticles, due to their wide range of antimicrobial behavior and chemical versatility [66]. ZnO-NPs are non-hazardous to human cells at certain concentrations. This property of ZnO has prompted researchers to examine its antibacterial and antioxidant effects [67]. CuO NP has some applications in biomedical and engineering fields. Biosensors, photodetectors, nanofluids, and wastewater treatment are only a few of the applications [68].

Copper oxide nanomaterials have sparked tremendous interest, due to their unique properties across all metal oxides, as well as their low cost, high accessibility, and long-term applications, such as antimicrobial properties [69]. Since copper is more vulnerable to oxygen than gold and silver, the oxide phases are more thermodynamically stable. Nevertheless, the presence of copper oxides on the surface of nanoparticles prohibits their use in electronics and biological fields [70]. AuNPs are a type of nanoparticle that is widely used in medicine, for medical and therapeutic purposes [71]. Significant AuNP properties, such as special optical, biocompatibility, physicochemical, functional versatility, flexible nanoparticles, controlled dispersion, heavy drug density surface area, stability, and non-toxicity, make them an efficient nanocarrier within drug delivery systems [42]. 

### Mechanism of Antimicrobial Activity of Metal Nanoparticles and Antibiotics

The antibacterial activity of NPs has been suggested to include contact with bacterial cells, thereby dignifying them, as well as moving NPs that interfere with cellular functions and metabolic pathways across the bacterial membrane, eventually leading to cell death [39]. The antimicrobial activity of nanomaterials is primarily based on the following three mechanisms: disruption of the cell membrane; release of heavy metal ions that would interact between proteins, resulting in a loss of protein structure, thereby hindering or killing organisms; and the development of reactive oxygen (ROS), an important reaction that kills RNA, proteins, and DNA, thus killing microorganisms [29]. Table 1 illustrates the antimicrobial nanoparticles and a certain mode of action.

**Figure 5:**
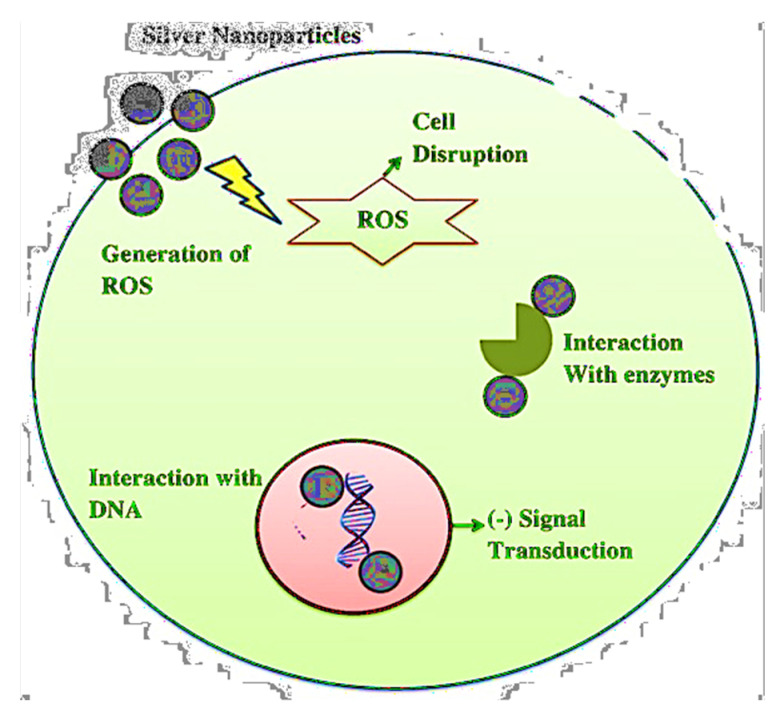
The modes of action of AgNPs composite against microorganisms.

Metal nanoparticles are antimicrobial agents with a wide spectrum of operations. These metal ions are thought to form tight coordination bonds with the N, O, and S atoms in biomolecules, as well as amines, phosphates, protein thiols, and DNA [79]. Although silver nanoparticles have strong antimicrobial properties, their antibacterial function has yet to be fully understood. Membrane destruction is one of AGNPs’ key mechanisms of action on microorganisms [82]. By anchoring the bacterial cell wall, silver nanoparticles induce structural changes in cell membranes, including changes in cell membrane permeability and cell death, as explained in Figure 5. Since nanoparticles primarily collide with the cell’s surface, pits form, in which the nanoparticles accumulate [83]. The interaction of silver nanoparticles with DNA causes the replication of DNA to be disrupted, resulting in the death of microorganisms. The nanoparticles dephosphorylate the peptide substrate found on Gram-negative bacteria’s tyrosine residues, inhibiting signal transduction, thus restricting bacterial growth. However, metallic nanoparticles (MNPs) have antibacterial properties, due to their high surface-to-volume ratios, which are normally associated with enhanced ROS production, including free radicals [84].

## 6. Cytotoxic Activity of the Metal Nanoparticles and Antibiotics Complex

Numerous NP–AB combinations, especially those from the largest critical class of antimicrobial agents, have been studied to enhance and recover the antibacterial effect of ineffective ABs (quinolones, carbapenems, lincosamides, penicillin, sulfonamides, glycoprotein, nitrofurans, tetracyclines, macrolides, polymyxins, including aminoglycosides), focusing mostly on the WHO (2016–2018) list [45]. In addition to metal-based NPs, metal oxide NPs (MO-NPs) have been widely used in laboratory antibacterial and anticancer research [85]. Silver nanoparticles interfere with the cell surface’s thiol protein groups, disrupting microbial oxygen levels and contaminant transport through the cell membrane [86]. Metal nanoparticles are becoming more interested in silver because it has distinct or changed properties, based on various aspects, such as morphology and size distribution. Silver nanoparticles have long been recognized for their antimicrobial properties, which have rendered them extremely useful in medicine [87]. Antibiotic nanomaterials have been successfully used to control infection in some therapeutic systems. Copper and its various nanomaterials have been used as antibiotics in medical care applications, to combat infection. Clay’s antibacterial properties have long been known, and with the advent of silver nanoparticles, its use is increasingly expanding. Ag NPs have been shown to have antibacterial and antifungal properties [82]. They were used as appropriate nanostructures in site-directed radiation therapy, the development of highly advanced electrochemical biosensors, the delivery of organic compounds (such as genes and proteins), as well as antibiotics, and the catalysis of chemical reactions [83]. Antimicrobial drugs have various modes of action, based on factors such as systematic conformity and adherence to the particular active site [84]. Drug-resistant factors are spread by transposable elements, through genes, and from one microorganism to the next, through various genetic mechanisms [88]. The biomedical applications of inorganic nanotechnology include diagnostic techniques, nano drugs, and delivery systems, and biomedical implants [89]. The conditions for using Fe_3_O_4_-NPs as a drug delivery mechanisms were investigated, to determine the best state for using Fe_3_O_4_-NPs in drug delivery. In vitro research has shown that Fe_3_O_4_-NPs have positive results for cancer cell therapy in some studies. Silver ions can kill microorganisms by destroying the cell membrane and preventing DNA replication [90]. Many studies show that nanotechnology can provide very useful methods for the delivery of drugs, and that this can be achieved with nanomaterials that need few adaptations and modifications, in terms of biopolymer decoration [91]. Nature-inspired, lipid-based, biopolymer-based, special equipment-based, and flexible nanocarrier machinery are the five types of nanocarrier structures [92]. In the fight against antibiotic resistance, dual drug delivery strategies that include antibiotics that target multiple pathways are the most researched and successful alternatives to multidrug formulations. Nanoparticles (NPs) are being used as a replacement for drugs to destroy bacteria, and their use is growing by the day. Implant products with antimicrobial composites, antimicrobials to minimize infection, which improves wound healing techniques, and antibiotic delivery systems are examples of biological activities [93].

## 7. Conclusions

Drug dosage and toxicity can be reduced using nanoparticles. Antibiotic use is the primary cause of antibiotic resistance in microorganisms, since it helps them to alter their genotype, resulting in multidrug strains. Due to its modern and lucrative applications, emerging nanotechnology is a significant aspect of science that is considered the most important technological breakthrough in recent years. Currently, metal nanoparticles as nanocarriers are being attained. Because of their wide-spectrum applications and higher compatibility with bioactive components, they have attracted a lot of interest from scientists. As a consequence of their ability to prevent the growth of bacteria, nanocomposites have been shown to have significant antibacterial, antifungal, antiviral, and antiparasitic efficacy in the battle against antibiotic resistance in microorganisms. Therefore, metal nanoparticles can be used as an antibiotic carrier to lower dose intake, minimize toxicity, and decrease the chances of resistance development. 

## Figures and Tables

**Table 1:** The antimicrobial properties of nanoparticles and antibiotic composite.

Source of Nanoparticle	Antibiotic	Resistant Bacteria	Mode of Action	References
Ag NPs	Ofloxacin	*Pseudomonas aeruginosa*	Blocking of efflux pump	[72]
Ag NPs	Tetracycline	*Staphylococcus aureus* and *Escherichia coli*	Synergistic reaction with exanimated antibiotic	[73]
Ag NPs	Teicoplanin	*Streptococcus pneumoniae*	ROS death of mediated cell	[74]
Ag NPs	Ampicillin	*Pseudomonas aeruginosa* and *Escherichia coli*	Synergistic action with antibiotic	[75]
ZnO NPs	Ampicillin	*Klebsiella pneumoniae*	ROS rupture of mediated cell wall	[76]
ZnO NPs	Methicillin	*Staphylococcus aureus*	Enzymatic inhibition	[77]
Au NPs	Vancomycin	*Staphylococcus aureus*	Synergistic action with exanimated antibiotic	[78]
Au NPs	Methicillin	*Staphylococcus aureus*	Photoinactivation and generation of ROS	[79]
TiO_2_ NPs	Methicillin	*Staphylococcus aureus*	Degradation of protein	[80]
TiO_2_ NPs	Multidrug resistant	*Escherichia coli*	Cell wall rupture and generation of ROS	[81]

## Data Availability

Data sharing is not applicable to this article.
